# Evaluation of Global Health in Master Swimmers Involved in French National Championships

**DOI:** 10.1186/s40798-015-0021-0

**Published:** 2015-06-10

**Authors:** François Potdevin, Gilles Vanlerberghe, Gautier Zunquin, Thierry Pezé, Denis Theunynck

**Affiliations:** 1 Univ. Lille, EA 7369 - URePSSS - Unité de Recherche Pluridisciplinaire Sport Santé Société, F-59000 Lille, France; 2 Univ. Littoral Côte d’Opale, Département STAPS, F- 62100 Calais, France; 3 Univ. Littoral Côte d’Opale, EA 7369 - URePSSS - Unité de Recherche Pluridisciplinaire Sport Santé Société, F-59144 Dunkerque, France

## Abstract

**Background:**

Swimming is often recommended as a means of increasing physical activity and gaining health benefits. The present study examined the psychological, social, and physical health states in competitive swimmers engaged in long-term training.

**Methods:**

The study took place during the 4 days of the French master championships in France in 2011 (from 10 to 13 March). Global health parameters were evaluated and compared with general values reported in studies aiming to describe health standard values in France or Europe. All swimmers selected for the event were invited to participate in the study. Setting questionnaires concerning mental and social health (short form 36), physical activity (International Physical Activity Questionnaire), and medication were administered. Peak expiratory flow (l.min^−1^) was measured, and body mass index (kg.m^−2^) was calculated from height (m) and body mass (kg). Prevalence of overweight and obesity was calculated by age and gender. Chi-squared tests were used to compare prevalence of overweight and obesity between participants and reference values. Short form 36 scores and physical activity (met.h.week^−1^) were compared with reference values by single *t*-tests. Two-way ANOVA was used to compare peak flow results with standard values. The level of significance was set at *p* < 0.05.

**Results:**

Out of 1554 master swimmers, 490 participated in this study (rates of participation = 44.8 and 23.5 % for females and males, respectively). Data showed inequality of health indexes as compared with reference values, despite a significantly higher level of physical activity including swimming activity. The prevalence of obesity was significantly lower (*p* < 0.05), and peak expiratory flow values were higher among female swimmers (from 7.6 to 17 % higher according to the age, *p* < 0.05). Perception of vitality was significantly higher for all female swimmers and the older age groups of male swimmers (*p* < 0.05). Perception of bodily pain indicated significantly lower scores for swimmers than the reference values (*p* < 0.05). Significantly lower prevalence of swimmers taking medication was noted in comparison with the French national values (*p* = 0.012).

**Conclusions:**

Compared with reference data from various sources, master competitive swimmers used less medication, had lower rates of obesity in most age groups, had greater peak expiratory flow values suggesting improved lung function, had higher levels of total physical activity, and had more favorable scores for various subscales of the SF-36. The results argue in favor of competitive swimming for its positive effects on health outcomes.

**Key Points:**

- French master swimmers involved in national championships have many positive health outcomes in terms of weight management, respiratory function, and vitality. However, the very high physical activity level in this competitive context did not result in significantly better levels on all parameters in comparison with their national counterparts (pain perception, social, emotional, and mental health).
- Positive health outcomes were higher for female master swimmers in terms of weight management, respiratory function, and vitality.
- The fact that these benefits were not linked with medication consumption could be interesting in an economic context aimed at controlling expenditures on health. Based on these data, we can argue for promoting this form of physical activity across the lifespan.

## Background

Regular physical activity throughout the lifespan improves health and decreases the prevalence of various chronic diseases and disorders [1, 2]. Accordingly, many programs to promote physical activity are being conducted around the world. The consensual recommendations state that all adults should carry out some degree of moderate or intense physical activity, preferably on a daily basis [3–5].

Among the possible kinds of physical activities, various authoritative groups and researchers often recommend swimming as a means of increasing physical activity and gaining health benefits. Swimming has proven to have multiple positive effects in the areas of the prevention and treatment of cardiovascular disease [6, 7], on cardiorespiratory fitness [8, 9], and on anthropometric measures such as body weight, fat distribution, waist circumference, body mass index [10, 11], and blood pressure [12]. Considered as an aerobic activity, swimming has been reported to have emotional state benefits among healthy people and clinical populations on anxiety depression, moods, or self-esteem [13, 14]. Despite studies showing that extensive training in swimming sometimes has detrimental effects on the respiratory system due to the chloramine context [15], and injuries involving the shoulders, spine, or blood pressure [16, 17], swimming is recognized over the world as an effective way to promote health.

Most studies aiming to assess the health benefits of swimming have focused on either physiological or psychological parameters. Experiments are often carried out in the short term (from 6 weeks to 1 year), which prevent them from measuring the long-term impact on overall health. According to the World Health Organization (WHO), the concept of health has to be defined as “a complete state of mental, physical, and social well-being” [18], suggesting that a multi-level analysis is needed to study the health benefits of swimming. Studies of overall health in a specific sample must therefore use several health indexes measuring different domains of health. In the case of a large sample, the health indexes used have to be relevant and easy to administer or measure at a given time. The body mass index (BMI; kg.m^−2^) is a physical health parameter for assessing excess adipose tissue, which is associated with increased risks such as type 2 diabetes, cardiovascular disease, high blood pressure, and dyslipidemia, as well as certain types of cancer [19]. Spirometric lung function measurement is commonly used for the diagnosis, assessment, and management of lung diseases [20]. This measure could be especially relevant for swimmers who practice in a chloramine environment. The short form 36 health survey questionnaire (SF 36) is widely used to evaluate several aspects of health related to physical mobility, emotional well-being, social life, and overall well-being [21].

Competitive master swimmers appeared to be interesting subjects for studying the health benefits of swimming because they spend more time training than the average sedentary aging person and allow one to test for the long-term effects of swimming. This population can be defined as adult swimmers, older than 25 years old, engaged in a regular training, and participating in several competitions per year. Dionigi [22] suggested that older people who compete in sports resist the dominant negative stereotypes associated with aging and feel empowered to live a fulfilling and healthy life. Few studies have aimed to assess the health impact of competitive training among master swimmers. Vaccaro et al. [23] showed a significantly lower percentage of fat in female master swimmers ages 20 to 70 as compared with untrained women. Walsh et al. [24] found a lower prevalence of obesity in master swimmers involved in world swimming championships. Guthrie et al. [25] noted that master swimmers had a lower prevalence of high blood pressure and took less medication to manage it than their national counterparts. Additionally, the results showed that master swimmers suffering from high blood pressure considered themselves healthier. Using the same methodology, Erickson and Guthrie [26] found fewer physically and mentally unhealthy days and fewer inactive days due to health problems among master swimmers.

The hypothesis of this study was that with regular and serious engagement in swimming training over a long period of time at older ages, French competitive master swimmers (FCMS) will have better health indexes than reported in studies aiming to describe standard population in France or Europe. The aim of this study was to analyze the physiological and psychological health indexes of FCMS in conjunction with those of comparative general populations to gain a better understanding of the links between swimming, participation in competitive events, and health benefits.

## Methods

### Study Design

The research hypotheses were examined by comparing health parameters of FCMS corresponding to the physical, physiological, and psychological domains to values reported in studies aiming to define standard ones in the French or European population. Physical activity was quantified by differentiating swimming and other activities to avoid effects of any physical activity other than swimming. In order to avoid effects of medication on health parameters, prevalence of swimmers taking medication was measured and compared with national French values.

### Setting

The study took place during the 4 days of the French master championships in France in 2011 (from 10 to 13 March). During the event, a team of five researchers and ten students had two rooms available in the aquatic center to administer questionnaires. Firstly, swimmers were invited to room 1 where questionnaires pertaining to mental health, physical activity, and medication consumption were administered. Then, they were asked to come to room 2 where height (m), body mass (kg), and peak expiratory flow (PEF; l.min^−1^) were measured. These rooms were attached to the warm-up and recovery pool.

### Participants

All of the 1554 swimmers selected for the event were eligible for the study. They were invited via posters, press conferences, website of the event, and speakers to participate in the study. Because of the research team’s location near the warm-up and recovery pool, it was easy to communicate about the main objective and the design of the study with the swimmers.

At their convenience during the event, swimmers were encouraged to meet the research team. They were asked to participate in the protocol when they had at least a break of 4 h between two races in order to limit stress effects due to the races.

All procedures followed were in accordance with the ethical standards of the responsible committee on human experimentation (institutional and national) and with the Helsinki Declaration of 1975, as revised in 2008. Informed consent was obtained from all patients for being included in the study. Ethics consent was granted through the French National Committee on data processing and freedoms (number 1487869v0).

### Data Collection and Variables

In the first room, one experimenter for four swimmers was present to supervise the written responses to questionnaires. The experimenter invited participants to ask questions in the case of lack of clarity and verify that all items were fully answered. Mental health and social health were evaluated via the SF 36 survey [21]. Seven of the current eight multi-item variables were measured: social functioning, role limitations due to physical problems, role limitations due to emotional problems, mental health, energy and vitality, pain, and general perception of health. Each item was scored out of a total of 100. The higher the score, the better the health parameter.

Physical activity was measured by using the long last 7 days self-administered version of the International Physical Activity Questionnaire (IPAQ) [27]. Each activity related to the workplace, transportation, housework, leisure time physical activity (LTPA), and time spent sitting were measured in duration and frequency to calculate metabolic equivalent tasks per week (MET-h.week^−1^) based on Ainsworth et al.’s proposal [28]. Concerning LTPA, recreation and sports, subjects were asked to not include swimming in this category. Swimming activity was measured in terms of the kind of weekly training and intensity level. Distance was calculated in MET-h.week^−1^, using the same scale. This differentiation between LTPA with and without swimming activity allowed evaluation of the ratio of swimming training on overall physical activity.

Medication consumption was taken into account by asking whether medical treatment in relation with categories found in French national reports was undergone: disease of bones and joints, cardiovascular disease, respiratory and ear noise and throat disease, digestive disease, and mental illness [29–35]. Ophthalmological problems and other reasons for taking medication were not measured in this study. Percentages of master swimmers taking the different types of medication were quantified.

In room 2, body mass (kg) and height (m) were directly measured by an experimenter because self-reported data gave underestimated values [36]. Body mass index (kg.m^−2^) was used to measure overweight and obesity. Prevalence of overweight and obesity in FCMS was calculated according to the WHO cutoff, that is, 25 ≤ BMI < 30 kg.m^−2^ and BMI ≥30 kg.m^−2^, respectively [37].

Lung function was assessed in PEF (l.min^−1^) for all subjects who had not smoked or used inhalers 1 h prior to the test. One of the experimenters demonstrated the correct manner for performing the test in a standing position. Subjects were observed while they made three trial attempts in order to detect faulty technique. Recovery of 30 s between trials was respected to avoid respiratory fatigue. Once they were able to perform the test correctly, they were asked to exert as much effort as possible. The highest value reached on three correct and reproducible tests (difference lower than 40 l.min^−1^) was recorded.

### Limiting Bias and Missing Data

In order to check the representativeness of the convenient sample, proportions by gender and age were compared with the rates of all swimmers selected for the event at the end of the third day of the competition. During the last day of the event, speakers encouraged specific age and gender low-participation classes to participate in the study. They were encouraged to participate in the study only when there was a break of 4 h between two races to limit stress or fatigue due to the events. Finally, the presence of a supervisor during the data collection by questionnaires allowed avoidance of missing data and lack of clarity of the questions.

### Statistical Methods

Standard statistical methods were used to calculate the means and standard deviations of the data. Normal Gaussian distribution and homoscedasticity were verified using Shapiro-Wilk’s and Levene’s tests. Age and gender classifications of FCMS were adjusted according to age classifications used in reference studies. For BMI and PEF results, ages were classified according to 25–29, 30–39, 40–49, 50–59, 60–69, and superior than 70 classes. For physical and SF 36 results, ages were classified according to 25–34, 35–44, 45–54, 55–64, and superior than 65 classes.

Representativeness of the sample was checked by two chi-squared tests in order compare the sample’s rates by age of each gender with rates in the whole population selected for the event. Prevalence of overweight and obesity in FCMS was compared with results obtained by Charles et al. [38] in the year 2006 in a French sample of 22,374 people using the chi-squared test. The authors kindly sent us the proportion by age and gender in order to compare with our data. Short form 36 scores were compared with values from Jenkinson et al. [39] using a single-sample *t*-test. Cronbach alpha coefficients were calculated for each section (social functioning, role limitations due to physical problems, role limitations due to emotional problems, mental health, energy and vitality, pain, and general perception of health). Peak expiratory flow (l.min^−1^) scores were compared with predicted values resulting from Nunn and Greg’s equations [40] using a two-way ANOVA (age-gender). International Physical Activity Questionnaire scores were compared with results from Rütten and Abu-Omar’s study [41] using a single-sample *t*-test. The prevalence of FCMS taking medications for each kind of disease was compared with French national references (bone and joint [30], cardiovascular [31], respiratory, ear noise and throat [34], digestive [35], mental illness [29]) using Wilcoxon’s test. For all tests used, the significance level was set at 0.05. Statistica software was used to perform statistical procedures.

## Results

Four hundred and ninety master swimmers (227 females and 263 males) participated in the study. The rates of participation for female and male and swimmers were 44.8 and 23.5 %, respectively. Chi-squared tests by age classes between samples of participants and all swimmers selected for the event showed no significant difference (*χ*^2^ = 5.26, *p* = 0.38; *χ*^2^ = 5.02, *p* = 0.41; for female and male swimmers, respectively). The age range was 25 to 95 years (45.9 ± 13.2 for women and 45.6 ± 12.9 for men), and the mean number of years of swimming training was 21 ± 13.5. Demographic description of the participants is presented in Table 1.

**Table 1:** Demographics of the participants (height (cm), body mass (kg), distance training a week (km), years of training in swimming, and years competing in master championships) by age group and gender

Age (years)		Height (cm)	Weight (kg)	Distance training per week in 2010	Years of training in swimming	Years competing in master championships
25–29	Females (*n* = 24, *N* = 71)	165 ± 0.1	63 ± 9.0	9.04 ± 4.9	15.15 ± 4.77	2.29 ± 1.1
	Males (*n* = 22, *N* = 99)	181 ± 0.1	77.3 ± 9.5	9.4 ± 4.9	15.7 ± 5.1	2.5 ± 1.3
30–39	Females (*n* = 64, *N* = 167)	167 ± 0.1	65.8 ± 13.0	8.0 ± 6.4	18.3 ± 7.5	5.6 ± 3.7
	Males (*n* = 59, *N* = 292)	180 ± 0.1	79.3 ± 9.1	9.9 ± 6.6	18 ± 7.7	6.8 ± 6.6
40–49	Females (*n* = 83, *N* = 176)	164 ± 0.1	63.3 ± 10.5	8.6 ± 4.5	18.6 ± 10.4	8.4 ± 6.0
	Males (*n* = 64, *N* = 286)	178 ± 0.1	79.6 ± 9.6	9.8 ± 5.2	20.3 ± 10.4	8.6 ± 7.7
50–59	Females (*n* = 57, *N* = 112)	164 ± 0.1	62.9 ± 7.8	9.1 ± 5.6	21.8 ± 14.6	12.8 ± 9.4
	Males (*n* = 45, *N* = 147)	177 ± 0.1	81.8 ± 10.6	10.4 ± 6.4	21.2 ± 14.2	12.0 ± 10.4
60–69	Females (*n* = 23, *N* = 48)	161 ± 0.1	62.6 ± 9.6	7.8 ± 4.63	27.2 ± 14.0	16.3 ± 11.8
	Males (*n* = 27, *N* = 91)	173 ± 0.0	75.6 ± 6.5	9.8 ± 5.01	33.2 ± 17.8	16.5 ± 16.6
>70	Females (*n* = 12, *N* = 16)	160 ± 0.0	68.6 ± 11.1	7.1 ± 5.03	46.3 ± 24.5	26.1 ± 12.4
	Males (*n* = 10, *N* = 49)	171 ± 0.1	73.4 ± 9.1	9.3 ± 5.52	41.7 ± 21.5	30.9 ± 25.0

*n* represents sample size and *N* represents total swimmers selected in French master championships by age and gender. Means and standard deviations were calculated for the sample size (*n*)

The physical activity levels of master swimmers are presented in Table 2. When swimming activity was included in the quantification, the physical activity of master swimmers was significantly higher than the reference values, whatever the age group (from 94 to 205 % according to the age group, *p* < 0.05). When swimming activity was excluded from the quantification, master swimmers had a significantly higher physical activity level in age groups 25–34 (*p* = 0. 002) and 45–54 (*p* = 0.002).

**Table 2:** Comparison of mean (±SD) MET-hours per week reported by individuals age 25 or older between master swimmers (with and without swimming) and European reference values [41]

Age (years)	Physical activity excluding swimming activity (MET-h.week^−1^)	Physical activity including swimming activity (MET-h.week^−1^)	European reference values (MET-h.week^−1^)
25–34	83 ± 154.4* (127.1 %)	111.5 ± 159.2* (205.1 %)	36.5
35–44	48.5 ± 93.7 (32.3 %)	79.7 ± 96.5* (117.4 %)	36.6
45–54	64.5 ± 110.7* (87.2 %)	96.5 ± 113.4* (180.1 %)	34.5
55–64	41.1 ± 62.8 (28.1 %)	75.1 ± 67.3* (134.3 %)	32.1
>65	24 ± 33.7 (−4 %)	48.8 ± 42.2* (94.9 %)	25.1

% represents the ratio between master values and the European reference values. MET corresponds to Metabolic Equivalent Tasks (MET-h.week^−1^)

*Significant differences with European normative data at *p* < 0.05

Results for the prevalence of participants categorized as overweight (25–29.99 kg.m^−2^) indicated no significant difference between FCMS and the French population (Table 3). Results for the prevalence of participants categorized as obese (≥30 kg.m^−2^) was significantly different for female swimmers in age groups 50–59 (1.8 % vs 16.6 %, *p* = 0.02) and 60–69 (3.8 % vs 17.7 %, *p* = 0.04) and for male swimmers in age groups 30–39 (1.6 % vs 9.3 %, *p* = 0.02) and 60–69 (4.2 % vs 20.9 %, *p* = 0.03).

**Table 3:** Prevalence of overweight and obesity by age group and gender for FCMS and French reference values [38]

	Females		Males	
Age	FCMS (%)	RF (%)	FCMS (%)	RF (%)
25–29 years				
Overweight (%)	11.5	13.6	20.0	19.7
Obesity (%)	3.8	7.2	4.0	5.2
30–39 years				
Overweight (%)	22.4	20.2	31.3	33.9
Obesity (%)	6.1	13.7	1.6*	9.3
40–49 years				
Overweight (%)	16.4	22.1	41.2	40.6
Obesity (%)	6.6	13.7	7.1	13.4
50–59 years				
Overweight (%)	28.1	29.2	45.6	45.1
Obesity (%)	1.8*	16.6	10.5	15.6
60–69 years				
Overweight (%)	34.6	33.6	54.2	47.5
Obesity (%)	3.8*	17.7	4.2*	20.9
70–79 years or older				
Overweight (%)	16.7	35.2	37.5	50.0
Obesity (%)	33.3	16.8	12.5	17.1

*Significant difference between French master swimmers (FCMS) and reference values (RF) at *p* < 0.05, according to overweight and obesity (25 ≤ body mass index < 30 kg.m^−2^ and body mass index ≥30 kg.m^−2^)

The results for PEF are presented in Table 4 and showed significantly higher PEF values for female FCMS from 25 to 60 years of age in comparison with the reference values (from 7.6 to 17 % higher according to the age classes, *p* < 0.05). Men between 40 and 50 years old only had significantly higher PEF scores than the reference values (5.6 %, *p* = 0.012).

**Table 4:** Peak expiratory flow (PEF; l.min^−1^): comparison between master swimmers and theoretical values by age group and gender [40]

	Females		Males	
Age (years)	Measured PEF (l.min^−1^)	Reference values (l.min^−1^)	Measured PEF (l.min^−1^)	Reference values (l.min^−1^)
25–29	493 ± 70*	441 ± 8	641 ± 81	627 ± 11
30–39	479 ± 66*	445 ± 8	643 ± 74	643 ± 13
40–49	482 ± 77*	429 ± 10	658 ± 74*	623 ± 32
50–59	476 ± 71*	407 ± 11	622 ± 99	598 ± 17
60–69	434 ± 89	377 ± 9	599 ± 107	540 ± 20
>70	305 ± 107	334 ± 17	529 ± 104	468 ± 42

*Significant difference between measured peak expiratory flow (PEF) and estimated PEF at *p* < 0.05

**Fig. 1:**
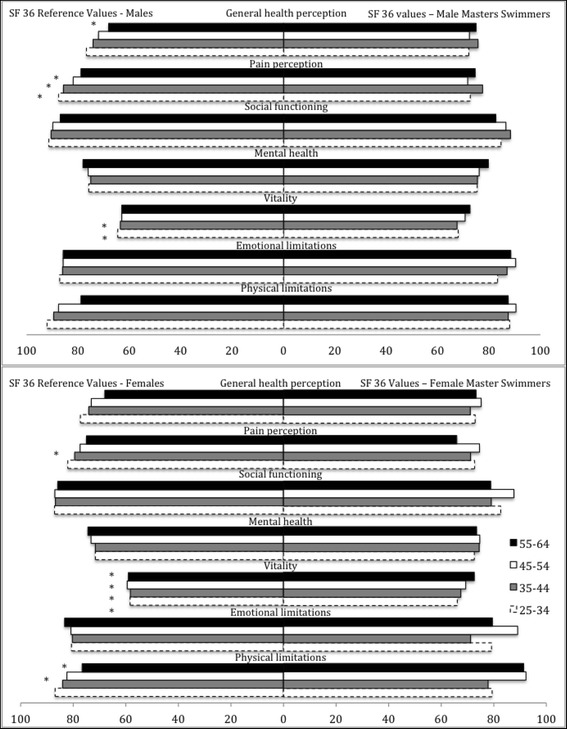
Short form 36 (SF 36) score comparison between French competitive master swimmers (FCMS) and reference values taken from Jenkinson et al. (1993). Alpha Cronbach coefficient values were 0.70 for physical limitations, 0.77 for emotional limitations, 0.72 for vitality, 0.78 for mental health, 0.75 for social functioning, 0.71 for pain perception, and 0.70 for general health perception. *Significant difference between FCMS and the reference values at *p* < 0.05

Perceptions of overall health in the physical, social, and psychological areas and Cronbach alpha coefficients are presented in Fig. 1. The results indicated significantly higher perceived health scores for female FCMS in terms of physical limitations (age groups 45–54 and 55–64, *p* = 0.002 and *p* = 0.003, respectively), vitality (all age groups, *p* < 0.05), and lower scores for pain perception (age group 35–45, *p* = 0.008). For male FCMS, the results indicated higher health scores for general health perception (age group 55–64, *p* = 0.03), for vitality (age groups 45–54 and 55–64, *p* = 0.001 and *p* = 0.001, respectively), and lower scores for pain perception (age 35 to 55, *p* < 0.05).

Prevalence of FCMS medication consumption is presented in Table 5. A significantly lower score was for medication consumption by swimmers in comparison with the national values for France (*p* = 0.012).

**Table 5:** Prevalence of medication consumption by disease category between participants and values reported in French national reports [29–31, 34, 35]

Drug consumption	Prevalence of master swimmers (%)		French national values (%)
Bones and joints	3.5		23
Cardiovascular	3.5		29
Respiratory, ear noise, and throat	4		10.2
Digestive	1.4		20
Mental	2		13
Probability		*	

*Significant difference (*p* < 0.05) in comparison across all disease categories between prevalence of master swimmers and French values

## Discussion

The aim of the present study was to determine whether master swimmers involved in French national championships exhibit better overall health when compared with the general population. The study assesses various health indexes to compare them with studies aiming to describe standard health values in French or European populations. The hypothesis was that competitive swimming increases all health indexes. Due to the large sample of participants (*n* = 490) with no significant difference in proportion with all swimmers selected for the event, this subsample can be considered as a representative of swimmers in French championships.

### Interpretation of the Main Results

As expected, overall physical activity was much greater in all age groups of master swimmers in comparison with the reference values in Europe [41]. Competitive swimming was associated with a large benefit in physical activity for older age groups (55–65 and over 65 years old) and for the 35–44 age group. For the older age groups, competitive swimming helped prevent the decline in physical activity with age [42]. It is worth noting that for two of our age groups (25–34 and 45–54), the physical activity of these master swimmers was higher than the reference values even when swimming was not taken into account.

The results for BMI (kg.m^−2^) indicated significantly lower prevalence of obese people among male and female swimmers in certain age groups. These results corroborate those obtained by Walsh et al. [24] showing a lower prevalence of obesity in master world game swimmers than in the national population of Australia. In our study, no significant difference in the prevalence of overweight people was noted between master swimmers and the French population, suggesting that swimming training is not linked to high weight loss. According to Gwinup [43], swimming is not the most effective form of physical activity for losing weight, which could explain the lower prevalence of obese people because of their high physical activity level, but not high enough to decrease weight to the point defined by the WHO (BMI <25 kg.m^2^). It is possible that, due to athletic activity, the French national master swimmers had a lower fat-to-lean body mass ratio than that of their national counterparts. Moreover, swimmers could benefit from a buoyancy advantage with fat deposits in certain regions of the body, and it is possible that swimmers may have tapered their diet and/or training to attain optimal body composition and physical performance for the competitive swimming event. The issue of causation must also be considered, that is, the question of whether participating in national French master swimming competitions promotes reduced obesity and lowers associated health risks or whether individuals with a lower BMI participate in master swimming championships by preference.

The PEF (l.min^−1^) results yielded significantly higher values for female master swimmers than for the theoretical values. For male master swimmers, no significant difference was noted, although all mean values were higher than the reference values. It is worth noting that the predictive values corresponded to a healthy level of lung function. Despite the chloramine environment, competitive swimming appeared to be effective at developing respiratory functions. These results corroborate the conclusions of several studies [44–46] showing the effect of swimming training on respiratory muscles. Swimming exercise affects lung volume measurements because the respiratory muscles of swimmers are used to develop greater pressure as a consequence of immersion in water during the respiratory cycle. This may lead to functional improvement in these muscles and a subsequent improvement in lung function. The fact that only female swimmers exhibited breathing performance above the predictive values could be due to the addition of this kind of exercise and a higher physical activity level than their European counterparts. However, more investigations are needed to explore respiratory functions because PEF (l.min^−1^) is only one parameter among the many indexes of respiratory health. In that way, the use of force expiratory volume in 1 s (FEV 1) and its ratio with force vital capacity (FVC) would be relevant in future research to assess both restrictive and obstructive lung disorders [47].

Short form 36 tests were conducted in order to evaluate psychological and social health. The results showed no significant difference between master swimmers and reference values for social functioning, emotional problems, mental health, and perception of overall health. Self-perception of vitality was significantly higher for all female swimmers and for the older age groups of male swimmers (from 45 to 65 years old). These results partially corroborate Acree et al.’s [48] study showing better scores on bodily pain, vitality, and social functioning for active as opposed to sedentary old people. Competitive swimming seems to impact vitality in the same way, but gave rise to the opposite effect on perception of bodily pain, with significantly lower scores for both female and male swimmers. Further investigation is needed to determine whether the higher self-perception of pain was due to a high physical activity level in an anti-gravity context or whether a sport involving a substantial proprioception of the body would increase the perception of pain.

Master swimmers took significantly less medication than their national counterparts (Table 5). In a study at population level, drug consumption had a positive and statistically significant effect on health status among middle-age individuals (ages 40 and 60 years) [49]. However, studies aimed at measuring the effects of drug consumption on population health have used macroscopic health indexes such as morbidity, life expectancy at different ages, or infant mortality [50, 51]. The results of the present study showed good health for master swimmers involved in French championships, with significantly lower intake of medication than their national counterparts (Table 5). From this point of view, competitive swimming at the national level appears to be a good way to improve health by increasing physical activity without managing health by means of medication.

### Limitations of the Study

As several cross-sectional studies have compliant samples, some results have to be taken into account with caution. The low rate of male master swimmer participation (24 %) could affect specific results concerning this gender. The free participation has limited master swimmers involved in a lot of races or selected only for 1 day of the event. It is difficult to consider that the male sample was representative of all male swimmers, even if the chi-squared test showed that the sample was not significantly different from the male master population. The second limit of this study was the old reference studies used to define standard values of health parameters. Unfortunately, our research in the standard baselines did not find more recent studies with standard values in France or Europe by age and gender. In this way, we had to adjust the age classes in our statistical methodology, which could affect statistical significance. Finally, the competitive context could affect some behavior such as anxiety, pain perception, or physical activity 7 days before the event, which could affect some health parameter values.

## Conclusions

It is a commonly held conception that adherence to an exercise regime improves all indexes of general health. The results of French master swimmers involved in national championships indicated unequal indexes to the reference values, despite their greater physical activity. Based on this data from French master swimmers, we can argue for promoting this form of physical activity across the lifespan, insofar as our participants had many positive health outcomes in terms of weight management, respiratory function, and vitality. The fact that these benefits were not linked with medication consumption could be interesting in an economic context aimed at controlling expenditures on health. However, the very high physical activity level in this competitive context did not result in better levels on all parameters. Indexes of the subjective perception of body pain were lower, and the impacts on social, emotional, and mental health were not statistically different. Further investigations are needed to explore in depth the outcomes of participating in a competitive sport that requires a high physical activity throughout life.
